# Druggists and Physicians

**Published:** 1880-05

**Authors:** 


### EDITORIAL DEPARTMENT.

"If thou hast truth to utter, speak;
And leave the rest to God."

DRUGGISTS AND PHYSICIANS.—The attitude of the druggist toward the physician is manifestly assuming, year by year, a more and more antagonistic form, pecuniarily; and unless a check is put upon counter prescribing, duplicating, and in some cases repeating almost indefinitely the recipes sent to them by the latter, an entire change will necessarily be made in the mode and manner of supplying patients with medicines by their attending physicians, and that too at an early day. There are other growing encroachments of the druggist upon the domain of the medical practitioner that require a check besides those just alluded to. But the most flagrant and undisguised violation of the comity that should be held sacred between the physician and the dispenser of medicines, is to be found in the almost constant prescribing by druggists for patients visiting their stores. In many cases they get nothing for their advice but the profit arising from the sale of the medicines they recommend. This is not always so, however. But in any case, would they deal fairly with their neighboring physician, the patient should be sent to him, and if unable to pay for both advice and medicine, we venture to say there are few physicians who would withhold a prescription in such a case because of the inability of the patient to pay for it. Such a course would inspire and perpetuate pleasant relation and confidence between the respective parties; for it must be obvious to any thoughtful person that the physician, though he should be only a recent graduate, must be more correctly acquainted with disease than a druggist who has not enjoyed the advantages of a medical education. Besides, in his arrogation of the functions of a physician he places the latter at an obvious disadvantage so far as the patient is concerned, even though he should not be able to pay for professional advice. These are some of the more prominent objections, but there are others which space will deny us the privilege of referring to at this time.

The next point to which allusion will be briefly made is that of repeating prescriptions by druggists; and whether the physician's property in it ceases *ipso facto* when he hands his prescription to a patient, and it becomes that of the latter or not, it can only extend to that special prescription and particular occasion unless otherwise stated or ordered at the time. This is evidently the common-sense view of the case, whether it is according to legal enactment or not; and by no kind of legerdemain can it become the property of the druggist at all. If not the property of the druggist nor patient, it is manifestly wrong for the druggist to compound or furnish the same medicines again to any one, even though requested so to do by the patient, except by the order or authorization of the physician from whom it emanated originally, unless the physician is compensated for it as at the first. This again would seem to be in harmony both with common-sense and common justice, likewise; and if so, such unauthorized renewals and refilling of prescriptions are in violation of the *lex non scripta*, at least, which every one innately understands who can appreciate the broad difference existing inherently betwixt right and wrong. We made a prescription for a lady in quite reduced circumstances on one occasion, and saw her no more for several months. Meeting her one day at the house of a well-to-do patient with whom she had been intimate in her better days, she said, after we had discharged our professional duty, and were about to leave, "Doctor, I have some good news to tell you. Do you recollect a prescription you gave me some months ago?" To which an affirmative answer was made. "Well, sir, it cured me completely in a week, after suffering for many years, and being under the treatment of some eminent physicians and the expenditure of hundreds of dollars; for my father spared no means that promised relief when his circumstances were good: and yet your prescription cost me but one dollar at the drug store." We were of course very glad to hear such news, and so stated, as about to leave, but she requested we should hear all about it, and said, "When I found myself well, I told Mrs. ——, an old friend, who had known of my sufferings for years and who herself was troubled to a considerable extent in the same way, that I had the bottle with the apothecary's name and label on it that cured me; and she could have the use of it, provided she would be very certain to preserve it carefully when she was done with it. She had it refilled and it cured her; and she recommended it to another lady affected similarly, and she was cured. And so on until the bottle was taken to the druggist and refilled for *nine* ladies besides myself, all of whom were either cured or greatly relieved by it; and we all feel so grateful for the remedy." We ventured to ask if all the ladies using the prescription were able to pay a physician's fee, and if so, they had utterly failed to make any pecuniary return to us from whom the prescription emanated; and we were nine dollars at least the loser by her philanthropy. But "she had not seen it in *that* light before." As we did not charge for the prescription originally, of course we got nothing at all, and the apothecary was benefitted to the extent of nine fillings of the bottle. This case is mentioned as an example only of hundreds of similar character that occur almost daily in every large community. But suffice it for the present.

